# Efficacy and Risks of Different Treatments for Oral Hyperpigmentation: A Systematic Review and Network Meta-Analysis

**DOI:** 10.3390/jcm12206567

**Published:** 2023-10-17

**Authors:** Ahoud Jazzar, Hebah AlDehlawi

**Affiliations:** Department of Oral Diagnostic Sciences, Faculty of Dentistry, King Abdulaziz University, Jeddah 21589, Saudi Arabia; haldehlawi@kau.edu.sa

**Keywords:** oral pigmentation, hyperpigmentation, treatment, management, laser, surgical stripping, bleeding, recurrence, pain, network meta-analysis

## Abstract

Background: Oral-pigmented lesions have raised aesthetic concerns, leading to multiple depigmentation techniques. This systematic review and network meta-analysis aimed to assess the efficacy of different treatments for oral hyperpigmentation. Methods: A computerized search was conducted on Science Direct, Medline via PubMed, Scopus, and Web of Science using the relevant keywords. English-language studies published between 2013 and 2023 that focused on patients with oral pigmented lesions subjected to different treatment modalities, such as laser or surgical intervention, were compared to determine their efficacy and safety profile. Data were analyzed using R software, applying frequentist models. Results: A total of 27 studies were included. In contrast to the CO_2_ laser, Er: YAG laser was linked to a higher risk of bleeding (RR = 2.73, *p* < 0.01), whereas the diode laser had the most favorable score in minimizing bleeding index (P-score = 0.86). In terms of lower risk and postoperative pain score (RR = 0.01, *p* < 0.01), the Er,Cr:YSGG laser had the most favorable result (P-score = 1.00). The Er: YAG laser demonstrated the highest probability of preventing recurrence (RR = 0.28, *p* < 0.01), followed by the diode laser (RR = 0.42, *p* < 0.01). Conclusions: The choice of treatment for oral pigmentation should be based on individual patient needs and the desired outcomes. The Er: YAG laser seems highly effective in preventing pigment recurrence, the diode laser emerges as a top contender in managing bleeding risks, and the Er,Cr:YSGG laser is particularly efficacious in managing postoperative pain.

## 1. Introduction

Oral pigmented lesions are considered uncommon lesions of the oral mucosa. Pigmentation can be classified into two main categories depending on the source of pigmentation: melanotic (intrinsic) or non-melanotic (extrinsic) pigment [1]. Melanin is a non-homogeneous endogenous pigment produced by melanocytes at the basal layer of the epithelium and transferred to upper layers via organelles without altering the normal architecture of the epithelium [2]. The color of the lesion depends on the amount, concentration, and level of melanin deposition, and ranges from light grey to brown or black pigmentation [3]. The deposition of melanin pigment is responsible for all melanocytic oral mucosal lesions. This includes diffuse patterns observed in physiologic pigmentation, smoking-associated melanosis, and pigmentation linked with systemic diseases and syndromes, as well as focal pigmentation found in conditions like oral melanotic macules [4]. Most of these lesions are diagnosed clinically, though in atypical cases, a biopsy may be conducted. Non-melanocytic oral lesions can appear as amalgam tattoos, heavy metal pigmentation, or pigmentation induced by tobacco and smoking [5]. Oral mucosal pigmented lesions can arise from drug-induced pigmentations, which result from the deposition of drug metabolites in the epithelium, potentially increasing melanin production [6]. In some instances, oral pigmentation can also be a side effect of radiotherapy [7].

In light of recent advances in aesthetic and cosmetic dentistry, oral pigmented lesions have become a significant aesthetic concern for many patients. Lesions appearing on the visible anterior parts of the oral mucosa, like the lips and gingiva, especially in patients with a high lip line, attract the most attention [8]. Various depigmentation techniques have been developed, tailored to the specific clinical presentation and the patient’s preferences [9,10]. Treatment options range from traditional surgical methods like excisional biopsy, gingival abrasion, or stripping, to more advanced procedures such as lasers, electrosurgery, and cryosurgery [11,12,13].

While surgical methods were once the predominant treatments for oral pigmented lesions, they often led to pain, patient apprehension, and recurrent pigmentation, necessitating extended postoperative care. As a response to these challenges, novel techniques emphasize less invasive and pain-free approaches. Laser treatment, for instance, has emerged as a popular alternative to traditional surgery [14]. Lasers produce concentrated light beams that are absorbed by the skin’s pigmented cells. The light energy is absorbed and transformed into heat, which specifically kills the pigmented cells that are being targeted and causes their elimination or decrease [15]. A photosensitizing agent is used in photodynamic therapy (PDT), which can be given intravenously or topically to the skin. A light source, usually a laser or LED, is used to activate the photosensitizer after a predetermined incubation period. Reactive oxygen species are produced as a result of this activation, which harm or kill the targeted cells, including pigmented lesions (selective photo-thermolysis) [16].

Various lasers, including CO_2_, Er:YAG, Nd:YAG, Er,Cr:YSGG, and diode lasers, have been employed to address these lesions. While laser treatments have demonstrated reliable outcomes, challenges like intricate parameter settings and potential recurrence persist [17]. Research indicates that, among these, diode lasers tend to have the longest depigmentation duration, especially in non-smoker patients [10]. Interestingly, Vitamin C, whether topically applied or injected intralesionally, has proven effective in treating dermal pigmentation. Its combination with conventional surgery for oral pigmented lesions has resulted in prolonged intervals before recurrence, suggesting a potentially cost-effective, simple, and minimally invasive depigmentation technique for the future [18]. This systematic review and network meta-analysis aimed to summarize the current evidence regarding the effectiveness of different approaches used in dentistry to manage different types of oral pigmented lesions.

## 2. Methods

### 2.1. Protocol and Registration

The Preferred Reporting Items for Systematic Reviews and Network Meta-Analyses (PRISMA-NMA) checklist and the Cochrane Handbook of Systematic Review and Meta-analysis were followed during the conduction of this study [19]. The protocol of this study was registered at PROSPERO International Prospective Register of Systematic Reviews (registration number: CRD42023397299).

### 2.2. Eligibility Criteria

We included the studies that met the following criteria: English language publication, studies published (in the last 10 years) between 2013 and 2023, randomized controlled trials on the treatment of hyperpigmentation, observational cohort studies, case series (≥10 cases), and cross-sectional studies. Animal studies, review articles, abstracts, case reports, and trials with irrelevant outcomes or with data that were not reliable for extraction were excluded. Patients with oral pigmented lesions to different treatment modalities such as laser or surgical intervention were compared to determine their efficacy and safety profile.

### 2.3. Information Sources and Search Strategy

A search was carried out via computer-based literature using four electronic databases: Science Direct, Medline via PubMed, Scopus, and Web of Science. The following keywords were used: (“Management” OR “Treatment” OR “Biopsy” OR “Laser” OR “Surgical” OR “Depigmentation” AND “Oral” AND “Pigmentation” OR “Hyperpigmentation” OR “Melanosis” OR “Melanese” OR “Hypermelanosis” OR “Hypermelanoses” OR “Staining” OR “Discoloration”). To enhance the sensitivity of the search strategy, the reference lists of the retrieved articles were hand-searched (records identified through other sources).

### 2.4. Study Selection

Two authors (HD and AJ) reviewed eligible studies; abstracts of identified publications using the search strategy were reviewed. After matching the title and abstract, any possibly eligible candidate articles were screened. Then, the article was fully reviewed to determine whether it fulfilled the inclusion criteria. Some articles were excluded after a review of the abstract or the full text if it was unrelated to the question’s topic. 

### 2.5. Data Items and Collection Process

Data extraction from eligible studies was performed by the authors (HD and AJ) independently. The data were recorded using a standardized spreadsheet, and it included authors, year of publication, study design, type of treatment, age and gender of population, sample size, pigmented lesion and its location, follow-up, and main outcomes, such as recurrence, risk of bleeding, bleeding index, and postoperative pain score assessed using visual analog scale (VAS). The bleeding index was assessed using the following scoring system: 1: no bleeding, complete homeostasis, 2: isolated bleeding points during surgery (mild), 3: moderate bleeding, but clear field, and 4: severe bleeding, difficulty in procedure.

### 2.6. Risk of Bias 

The evaluation of study quality and potential bias was conducted using the Risk of Bias 2 tool (ROB-2) developed by the Cochrane Collaboration. The domains studied involved a randomization process, deviations from intended interventions, missing outcome data, measurement of the outcome, and selection of the reported result. Each domain was meticulously evaluated to determine the extent of bias that could potentially influence study outcomes. A clear and structured approach was adopted to rate the risk of bias as either “low”, “some concerns”, or “high” for each individual domain, subsequently contributing to an overall judgment on the study’s risk of bias. For non-RCTs, we utilized the Risk of Bias In Non-randomized Studies of Interventions (ROBINS-I) tool to evaluate confounding, selection of participants, and other bias-inducing factors. Lastly, for cross-sectional studies, we employed the National Institutes of Health (NIH) Quality Assessment Tools.

### 2.7. Data Analysis

All data analyses were conducted using the R software. The frequentist model was applied using the “netmeta” package. The effect size was quantified as mean difference (MD) or risk ratio (RR), with corresponding 95% Confidence Intervals (CIs), and the outcomes were meticulously visualized through forest plots, net graphs, and net league tables. Initially, a fixed-effects model was employed, and in instances where heterogeneity was observed, the analysis was transitioned to a random-effects model to account for potential variability among the studies. In addition to assessing the overall heterogeneity using I², Q statistic, and its associated *p*-value, we conducted an evaluation of inconsistency within the network. This included the calculation of tau² and tau, contributing to a thorough understanding of the level of inconsistency present within the included studies. A *p*-value of less than 0.05 was considered statistically significant.

## 3. Results 

### 3.1. Study Selection

**Figure 1:**
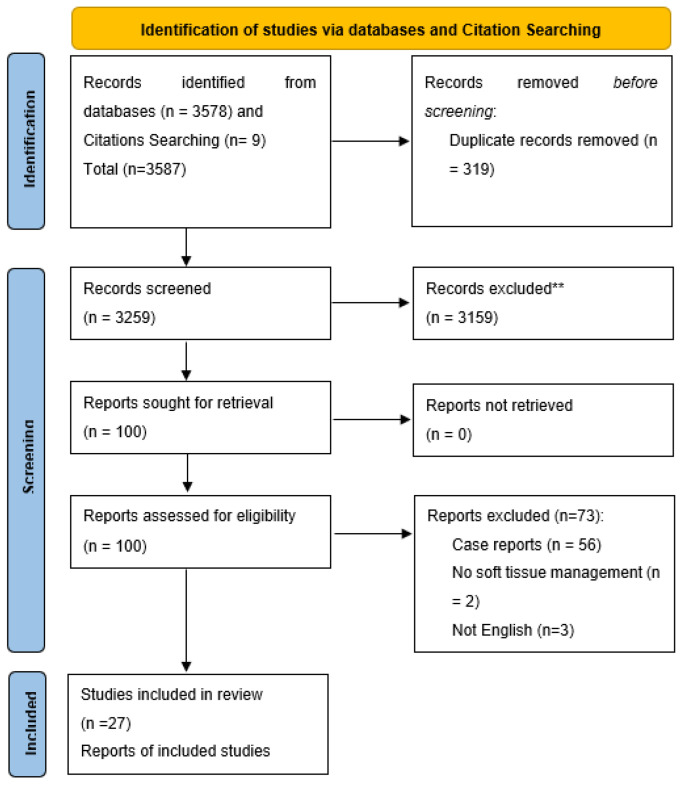
PRISMA flow diagram; ** After title and abstract screening.

The literature search of the selected databases and citation searching resulted in 3587 studies. After removing the duplicates (n = 319), a total of 3259 studies were included in the title and abstract screening. After excluding the ineligible studies (n = 3159), 100 full texts were screened. Out of these studies, only 27 studies were included in the qualitative and quantitative synthesis, as shown in Figure 1.

### 3.2. Characteristics of Included Studies 

The included studies in this review exhibit a diverse range of study designs and methodologies, including randomized controlled trials (RCTs), controlled clinical trials (CCTs), and cross-sectional studies. Interventions across the studies vary, from different types of lasers like the diode, CO_2_, Er:YAG, and Er,Cr:YSGG lasers, to surgical methods like stripping, and even novel approaches like intraepidermal vitamin C injections (mesotherapy). The sample sizes range from as small as 5 to as many as 60 participants, with ages spanning from 15 to 50 years. Most studies focus on physiological pigmentation, and the anatomical sites are largely confined to the upper and lower gingiva, although some studies also examine the lips. The follow-up periods range from a brief one week to as long as two years, offering a mix of short-term and long-term outcome assessments. Overall, the studies provide a comprehensive, albeit heterogeneous, landscape of treatment options for oral pigmented lesions, as shown in Table 1.

### 3.3. Risk of Bias

Out of the 16 included RCTs, six studies revealed a low risk of bias and 10 studies showed some concerns in terms of the randomization sequence and allocation concealment. Out of the six non-RCTs, two studies showed a moderate risk of bias, and the rest demonstrated a low risk of bias. Among the three case series studies, two studies included non-consecutive cases and non-comparable subjects. In addition, the three studies did not report detailed statistical methods. The sample size was not justified in the two cross-sectional and cohort studies. Supplementary File S1 summarizes the results of the risk of bias assessment.

### 3.4. Network Meta-Analysis

#### 3.4.1. Bleeding Risk

**Figure 2:**
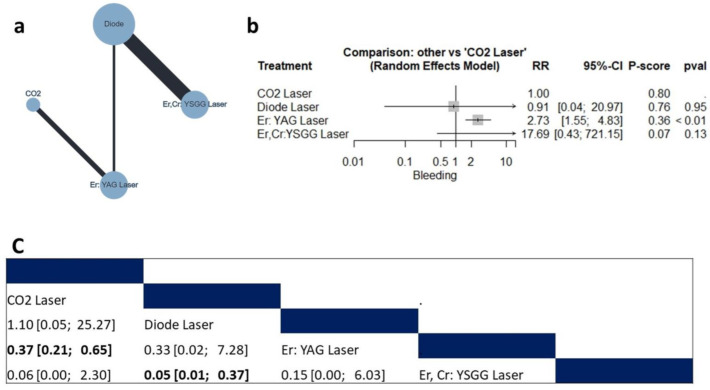
Network meta-analysis of bleeding. (**a**) Net graph. (**b**) Forest plot. (**c**) Net league table.

Four studies (n = 172 patients) reported data regarding bleeding risk [10,26,35,42]. The compared interventions were the diode laser, Er,Cr:YSGG laser, Er: YAG laser, and CO_2_ laser (Figure 2a). The fixed model effect estimate showed that compared with the CO_2_ laser, the Er: YAG laser was associated with a higher risk of bleeding (RR = 2.73, 95% CI: 1.55 to 4.83), as shown in Figure 2b. Additionally, the diode laser was associated with a lower risk of bleeding compared to the Er,Cr:YSGG laser (RR= 0.05, 95% CI: 0.01 to 0.37). On the other hand, there was no significant difference between the CO_2_, diode or Er,Cr:YSGG lasers (Figure 2c). The pooled data were homogenous (tau^2^ = 0; I^2^ = 0%; Q = 0.06; *p* = 0.80). There was no observed inconsistency between direct and indirect analysis. According to the ranking test, CO_2_ had the highest favorable score (P-score = 0.80).

#### 3.4.2. Bleeding Index

**Figure 3:**
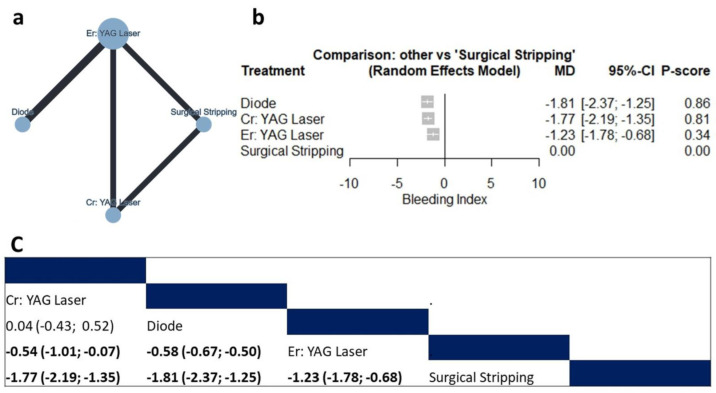
Network meta-analysis of bleeding index. (**a**) Net graph. (**b**) Forest plot. (**c**) Net league table.

Two studies (n = 63 patients) reported data regarding the bleeding index [20,38]. The compared interventions were the diode laser, Cr: YAG laser, Er: YAG laser, and surgical stripping (Figure 3a). The fixed model effect estimate showed that compared with surgical stripping, the diode laser (MD = −1.81, 95% CI: −2.37 to −1.25), the Cr: YAG laser (MD = −1.77, 95% CI: −2.19 to −1.35) and Er: YAG laser (MD = −1.23, 95% CI: −1.78 to −0.68) were associated with a significantly lower bleeding index, as shown in Figure 3b. Additionally, the diode laser and Cr: YAG laser were associated with a significantly lower bleeding index compared to the Er: YAG laser (MD = −0.58, 95% CI: −0.67 to −0.50, and MD = −054, 95% CI: −1.01 to −0.07 for the diode and Cr: YAG lasers, respectively. On the other hand, there was no significant difference between the diode laser and Cr: YAG laser (Figure 3c). There was no observed inconsistency between direct and indirect analysis. According to the ranking test, the diode laser has the highest favorable score (P-score = 0.86).

#### 3.4.3. Postoperative Pain Score

**Figure 4:**
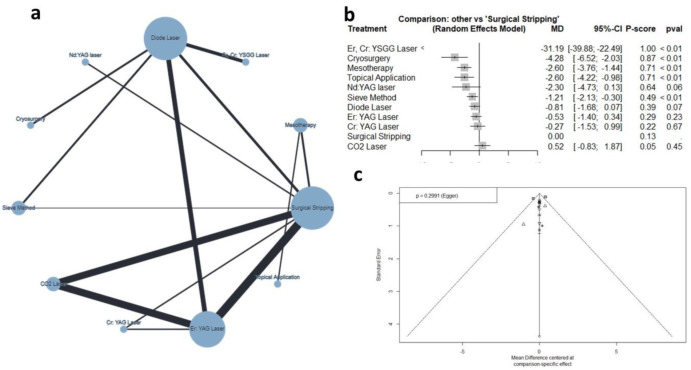
Network meta-analysis of postoperative pain score. (**a**) Net graph. (**b**) Forest plot. (**c**) Funnel plot.

**Figure 5:**
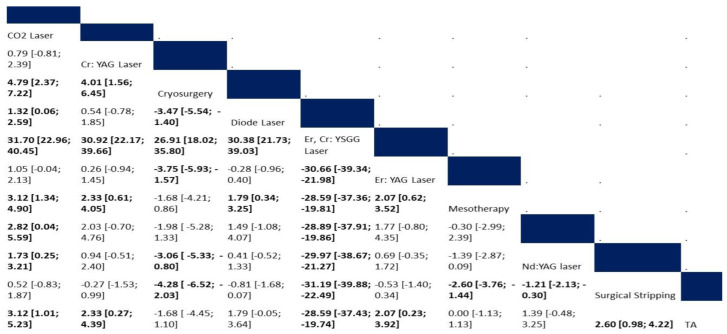
Net league of postoperative pain score.

Thirteen studies (n = 549 patients) reported data regarding the postoperative pain score [12,18,20,21,22,24,28,30,35,38,39,41,42]. The net graph summarizes the studied interventions (Figure 4a). The random effect model showed that compared to surgical stripping, the Er,Cr:YSGG laser (MD= −31.19, 95% CI: −39.88 to −22.49), cryosurgery (MD= −4.28, 95% CI: −6.52 to −2.03), mesotherapy (MD = −2.60, 95% CI: −3.76 to −1.44), topical applications (MD= −2.60, 95% CI: −4.22 to −0.98), and sieve method (MD= −1.21, 95% CI: −2.13 to −0.30) were associated with significantly lower pain scores, as shown in Figure 4b and Figure 5. The pooled data were heterogenous (tau^2^ = 0.27; tau = 0.52; I^2^ = 80.1%). The main source of heterogeneity was observed in the diode–Er: YAG laser comparison. There was no significant inconsistency between direct and indirect comparisons (Q = 1.91; *p* = 0.5907). According to the raking test, the best-performing intervention was the Er,Cr:YSGG laser, followed by cryosurgery, mesotherapy, and topical applications. According to the funnel plot and Egger’s test, there was no significant publication bias (*p* = 0.299), as shown in Figure 4c.

#### 3.4.4. Recurrence 

##### Risk of Recurrence

**Figure 6:**
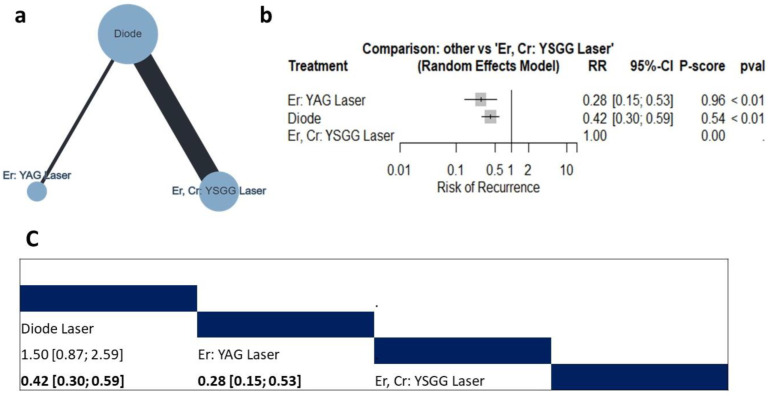
Network Meta-analysis of the risk of recurrence. (**a**) Net graph. (**b**) Forest plot. (**c**) Net league table.

Three studies (n = 132 patients) reported data regarding the risk of recurrence [10,26,35]. The compared interventions are described in Figure 6a. The random model effect estimate showed that compared with the Er, Cr: YSGG laser, the Er:YAG laser and diode laser were associated with a significantly lower risk of recurrence (RR = 0.28, 95% CI: 0.15 to 0.53 and RR = 0.42, 95% CI: 0.30 to 0.59, respectively), as shown in Figure 6b. Additionally, compared to the Er: YAG laser, the diode laser showed comparable efficacy (RR = 1.50, 95% CI: 0.87 to 2.59) (Figure 6c). The pooled data were homogenous (tau^2^ = 0.38; I^2^ = 32.5%). There was no observed inconsistency between direct and indirect analysis (Q = 1.48; *p* = 0.22).

##### Density of Repigmentation

**Figure 7:**
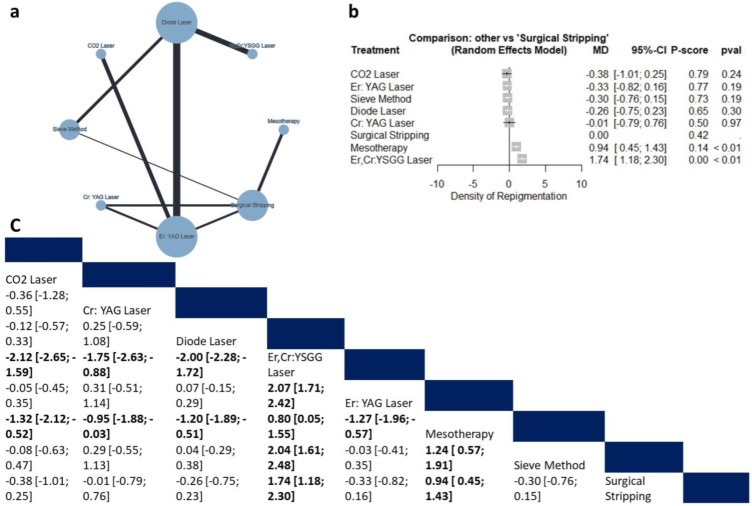
Network Meta-analysis of Density of repigmentation. (**a**) Net graph. (**b**) Forest plot. (**c**) Net league table.

Eight studies (n = 267 patients) reported data regarding the density of repigmentation. The compared interventions are described in Figure 7a. The random model effect estimate showed that compared with surgical stripping, mesotherapy and the Er,Cr:YSGG laser were associated with a significantly elevated density of repigmentation (MD = 0.94, 95% CI: 0.45 to 1.43 and MD = 1.74, 95% CI: 1.18 to 2.30, respectively), as shown in Figure 7b. Additionally, compared to the Er,Cr:YSGG laser, all interventions were associated with significantly lower density recurrence (Figure 7c), making the Er,Cr:YSGG laser the lowest effective intervention (P-score = 0.00). The pooled data were moderately heterogeneous (tau^2^ = 0.018; I^2^ = 50%). There was no observed inconsistency between direct and indirect analysis (Q = 4.1; *p* = 0.124). 

#### 3.4.5. Summary of the Study Findings

In this comprehensive network meta-analysis, we evaluated various interventions for oral pigmented lesions; multiple outcomes were studied, including bleeding, pain, and recurrence. The Er:YAG laser was found to be associated with a higher risk of bleeding compared to the CO_2_ laser, while the diode laser had a lower risk of bleeding than other laser types. The diode laser and Cr:YAG laser also significantly reduced the bleeding index compared to surgical stripping and the Er: YAG laser. Regarding postoperative pain, the Er,Cr:YSGG laser was associated with the lowest risk of moderate-to-severe pain, whereas the diode laser showed a significantly higher risk. In terms of recurrence, the Er,Cr:YSGG laser had the worst performance, with the highest density of repigmentation. The diode laser and Er: YAG laser were more effective in achieving the lowest risk of recurrence. The results were generally consistent across direct and indirect analyses.

## 4. Discussion

In this network meta-analysis, bleeding, pain, and recurrence were studied in relation to different therapies for oral pigmented lesions.

### 4.1. Bleeding

Our results demonstrate that the Er:YAG laser poses a significantly higher risk of bleeding compared to the CO_2_ laser but a significantly lower bleeding index compared to surgical stripping. This corroborates the findings of previous studies that have also found the Er:YAG laser to be associated with increased bleeding rates [24,38]. On the other hand, the diode laser was found to have a lower risk of bleeding than the Er,Cr:YSGG laser, and it also significantly reduced the bleeding index compared to surgical stripping and the Er:YAG laser. This is consistent with earlier studies that highlighted the hemostatic benefits of diode lasers [43,44]. The superior hemostatic properties of the diode laser can be attributed to its wavelength’s ability to penetrate deeper into soft tissues than that of the Er:YAG laser [45]. Additionally, the diode laser’s wavelength has a better balance than the Er:YAG laser between tissue removal and minimizing thermal damage, leading to reduced bleeding [46]. Furthermore, it was observed that the Er:YAG laser led to the significant dilation of blood vessels, which likely contributed to delayed gum healing and persistent bleeding post treatment. The mechanism of action of the laser, which quickly vaporizes the water content of the targeted tissues, is probably a cause of the phenomenon as well [24]. Similar to our findings, Moeintaghavi et al. reported no significant difference between the diode laser and CO_2_ laser in terms of bleeding incidence [47]. In contrast to various laser methods, surgical stripping had a remarkably higher bleeding index, corroborating earlier research [42,48,49,50]. The elevated risk of bleeding in surgical stripping could lead to the formation of minor hematomas, which subsequently take a longer time to convert into a fibrin layer. This thicker fibrin layer results in a slight delay in the healing process, especially in instances where the pigmentation is deeper [20].

### 4.2. Postoperative Pain

When it comes to postoperative pain, our findings indicate that the Er,Cr:YSGG laser is associated with the lowest risk of experiencing moderate-to-severe pain and the lowest postoperative pain score compared with other interventions. This finding was observed in several studies, such as Beşiroğlu-Turgut et al. [51], Bakhshi et al. [35], and Gholami et al. [20]. Suthprasertporn indicated that lasers in the Er,Cr:YSGG category result in reduced levels of pain compared to lasers outside the Erbium family. Furthermore, lasers possess the capability to seal off sensory nerve endings, contributing to lower pain perception [52]. Moreover, cryosurgery, mesotherapy, topical applications, and the sieve method were associated with significantly lower scores of postoperative pain compared to surgical stripping. Cryosurgery eliminates tissue by subjecting it to extreme cold through the use of cryogenic substances [8,53]. According to various studies, this technique often eliminates the need for local anesthesia, and is linked with reduced postoperative pain and bleeding [54,55]. However, cryosurgery has some limitations, such as postoperative swelling and challenges in regulating the depth of penetration [56]. Regarding mesotherapy and topical applications, the intensity of postoperative pain ranges from no pain to mild [18,39].

In contrast, surgical stripping and some laser techniques such as the CO_2_, Cr: YAG, Er: YAG, and diode lasers showed a significantly higher risk of inducing postoperative pain. Similar to our findings, a previous pairwise meta-analysis showed that there was no significant difference between surgical stripping and various types of laser, including diode, CO_2_, Cr: YAG, and Er: YAG, in terms of postoperative pain [57]. Adequate topical anesthesia is required in surgical stripping to manage the associated pain [58,59]. While lasers are effective in reducing pigmentation, they can cause discomfort due to the thermal energy they deliver to the tissues [21,37,49,60]. The level of pain experienced can vary based on individual pain thresholds, the type of laser used, and the depth of the pigmentation being treated [49]. This finding is an important consideration for patient care, as managing postoperative discomfort is a key factor in improving patient satisfaction and outcomes [61].

### 4.3. Recurrence

Regarding recurrence, the Er,Cr:YSGG laser and mesotherapy were found to be the least effective, showing the highest density of repigmentation post treatment. This is a critical finding, as recurrence often signifies treatment failure and may necessitate additional interventions. Likewise, Altayeb et al. showed that the repigmentation intensity and extensity were significantly higher in the Er,Cr:YSGG group than in the diode group at one year and two years post procedure (*p* < 0.05) [10]. In contrast to this finding, Beşiroğlu-Turgut et al. and Bakhshi et al. showed that Er,Cr:YSGG was associated with significantly lower pigmentation density and a higher percentage of no recurrence compared to the diode laser [35,51]. Several elements contribute to the variability in treatment outcomes for recurrence, including the type of treatment used, the duration of follow-up, assessment criteria for pigmentation, as well as genetic, ethnic, and hormonal factors [21]. The lower efficacy of Er,Cr:YSGG compared to other lasers could be attributed to the fact that the Er,Cr:YSGG laser has a lesser penetration depth, leaving melanocytes and melanin-containing keratinocytes intact in the basal layer. While the exact mechanism driving recurrence is not well-defined, the migration theory suggests that active melanocytes from surrounding pigmented tissue move to the treated areas, leading to recurrence [48]. All studied lasers, except for Er,Cr:YSGG, the sieve method, and surgical stripping showed better outcomes compared to mesotherapy. Both the diode laser and Er:YAG laser were more effective in lowering the risk of recurrence. This implies that these laser types could be more suitable for patients who are particularly concerned about the aesthetic outcomes of their treatment.

### 4.4. Clinical Implications

Clinicians should weigh the benefits and risks when deciding on the type of intervention for treating oral pigmented lesions. For instance, while the Er:YAG laser may result in higher bleeding, it performed relatively better in terms of recurrence. For patients with comorbid conditions where increased bleeding could be detrimental, such as those with hemophilia or patients on anticoagulant therapy, a diode laser may be the more suitable treatment option given its lower risk profile for bleeding. If postoperative pain is a major concern for the patient, the Er,Cr:YSGG laser could be the preferred option. It could also reduce the need for additional pain medications postoperatively, which is particularly important for patients who may have contraindications to certain analgesics. For individuals who are particularly concerned about the cosmetic outcome of the treatment, especially those with lesions in aesthetically sensitive areas, the choice between the diode laser and Er:YAG laser becomes relevant, as these options have shown better results in minimizing recurrence. The cost of these laser treatments can vary widely. Understanding the comparative effectiveness and risks associated with each can help clinicians make more cost-effective choices that do not compromise patient safety or outcome. Given the varying profiles of these laser treatments, a multidisciplinary approach involving dermatologists, oral surgeons, and even patient input might be beneficial. This can help in tailoring the treatment plans to individual patient needs and expectations. Finally, clinicians can use the evidence from this meta-analysis to better inform patients about what to expect from different treatment options, thereby aiding in shared decision-making.

### 4.5. Future Perspectives

While our study provides an insightful snapshot into the immediate outcomes associated with these treatments, the long-term efficacy and safety profiles remain less understood. Future studies should focus on long-term follow-ups to gauge durability and delayed complications. In addition, incorporating patient-reported outcome measures, such as quality of life or satisfaction scores, could provide a more holistic understanding of treatment effectiveness. Investigating the effectiveness of combining different laser types or using laser therapy in conjunction with other treatments, such as topical medications, could open new avenues for optimized patient care. Further research may also examine any geographical or ethnic variations in treatment responses, which would be valuable for clinicians treating diverse patient populations. Given the costs associated with laser treatments, future studies should also include cost-effectiveness analyses to guide healthcare policy and resource allocation decisions.

### 4.6. Limitations

While our study provides a comprehensive overview of the risks and benefits associated with different treatment modalities for oral pigmented lesions, we acknowledge that our study has some limitations. The number of included studies in some analyses, such as bleeding and recurrence, is relatively small. The observed heterogeneity in postoperative pain analysis is another limitation. Additionally, other important outcomes like wound healing, patients’ satisfaction, and costs were not evaluated due to the lack of data. 

## 5. Conclusions

Our network meta-analysis provides a comprehensive evaluation of the effectiveness and associated risks of various treatment options for oral pigmented lesions. Based on our findings, the choice of treatment for oral pigmentation should be based on individual patient needs and the desired outcomes. The Er: YAG laser seems highly effective in preventing pigment recurrence, the diode laser emerges as a top contender in managing bleeding risks, and the Er,Cr:YSGG laser is particularly efficacious in managing postoperative pain. These insights may serve as a guide for clinicians in making more informed decisions tailored to the individual needs and concerns of their patients. Further studies are required to evaluate the differences in terms of wound healing, patients’ satisfaction, and costs of these interventions.

## Figures and Tables

**Table 1:** Summary of included studies and patients.

Study ID	Study Design	Interventions	Sample Size	Age	Males	Pigmented Lesions	Location	Follow-Up
Altayeb et al., 2021 [10]	RCT	Diode 940 nm and Er,Cr: YSGG 2780 nm	60	21–43	22	Physiological pigmentation	Upper gingiva	2 years
Gholami et al., 2018 [20]	RCT	Er,Cr 2 settings/surgical stripping	36 arches	18+	12	Physiological pigmentation	Upper and lower gingiva	12 months
Hegde et al., 2013 [21]	CCT	Surgical stripping/carbon dioxide laser/Er YAG	35	18–50	15	Physiological pigmentation	Upper and lower gingiva	6 months
Ribeiro et al., 2014 [22]	RCT	Nd YAG/scalpel stripping	11	NA	NA	Physiological pigmentation	Upper anterior gingiva	6 months
Limpjaroenviriyak ul et al., 2020 [23]	RCT	QS 532 nm/LFQS 1064 nm both Nd YAG	30	20+	8	Multifactorial	Lip	4 weeks
Giannelli et al., 2014 [24]	RCT	Er YAG/diode lasers	21	18–40	10	Physiological pigmentation	Upper and lower gingiva	6 months
Tran et al., 2022 [25]	Cross-sectional	CO_2_ laser	38	20–24	19	Physiological pigmentation	Upper anterior gingiva	6 months
Ranj Arif et al., 2020 [26]	RCT	Er YAG 2940/diode lasers 940	20	18–35	16	Physiological pigmentation	Upper and lower anterior gingiva	6 months
Rohini Negi et al., 2018 [13]	RCT	Diode laser/ceramic soft tissue trimming bur	20	20–40	NA	Physiological pigmentation	Upper anterior gingiva	6 months
Stas et al., 2018 [27]	RCT	Er YAG (4 different settings)	40	18–50	NA	Physiological pigmentation	Upper and lower gingiva	6 months
Suryavanshi et al., 2017 [11]	Non-RCT	Surgical blade/ electrosurgery/ FGG/diode laser	40	NA	NA	Physiological pigmentation	Upper anterior gingiva	3 months
Houshmand et al., 2017 [28]	Non-RCT	Diode laser 940 (2 methods, sieve or conventional)	15	Mean 33	5	Physiological pigmentation	Upper and lower anterior gingiva	3 months
Roshannia et al., 2021 [29]	Non-RCT	Diamond bur abrasion/CO_2_ laser	12	18–40	NA	Physiological pigmentation	Upper and lower anterior gingiva	6 months
Surve et al., 2020 [30]	RCT	Surgical blade/sieve method diode laser	5	18–40	NA	Physiological pigmentation	Anterior gingiva	1 year
Hawwam et al., 2020 [31]	Case series	Surgical blade	20	23–30	11	Physiological pigmentation	Upper and lower gingiva	1 year
AlShoubaki et al., 2018 [32]	Cohort	Rotary diamond bur abrasion	34	19–45	10	Smokers’ melanosis	Upper and lower gingiva	6 months
Jokar et al., 2019 [12]	RCT	Cryosurgery/Diode laser 940	15	17–35	4	Physiological pigmentation	Upper anterior gingiva	1 year
Juliana et al., 2022 [33]	RCT	Blue M gel/Coe pack, both after the surgical blade	20	20–38	NA	Physiological pigmentation	Upper anterior gingiva	4 weeks
Koca-Ünsal et al., 2021 [34]	RCT	Surgical blade/Diode laser 810	16	23–41	8	Physiological pigmentation	Anterior gingiva	1 week
Bakhshi et al., 2018 [35]	Non-RCT	Er CR YSGG/diode	14	15–39	5	Physiological pigmentation	Upper and lower gingiva	6 months
El Shenawy et al., 2015 [36]	Case series	Diode 980	15	15–40	7	Physiological pigmentation	Upper and lower anterior gingiva	3 months
Kishore et al., 2014 [37]	RCT	Er YAG/CO_2_ laser	20	18–30	10	Physiological pigmentation	Upper anterior gingiva	6 months
Harb et al., 2021 [38]	RCT	Diode 980 nm/Er YAG	12	28.6 ± 7.8	7	Physiological pigmentation	Upper and lower gingiva	6 month
El-Mofty et al., 2021 [18]	RCT	Injection and topical vitamin C	20	18–40	4	Physiological pigmentation	Upper and lower anterior gingiva	6 months
Chaudhary et al., 2023 [39]	RCT	Surgical blade/intraepidermal injection of vitamin C	30	18–40	18	Physiological pigmentation	Upper and lower anterior gingiva	3 months
Yussif et al., 2019 [40]	Non-RCT	Vitamin C injection intraepidermal/surgical blade	30	18+	NA	Physiological pigmentation	Upper and lower anterior gingiva	9 months
Grover et al., 2014 [41]	Non-RCT	Surgical blade/diode laser 800–980 nm	20	15–35	11	Physiological pigmentation	Upper and lower anterior gingiva	3 months

RCT: randomized controlled trial; CCT: comparative clinical trial.

## Data Availability

Data available upon request from authors (ojazzar@kau.edu.sa).

## Supplementary Materials

The following supporting information can be downloaded at: https://www.mdpi.com/article/10.3390/jcm12206567/s1. Supplementary file S1 summarizes the results of the risk of bias assessment.
